# The Protective Effects of Buzui on Acute Alcoholism in Mice

**DOI:** 10.1155/2016/3539748

**Published:** 2016-01-17

**Authors:** Chen Chen, Da-Chao Wen, Shu-di Gao, Xiao-yu Hu, Cheng Yi

**Affiliations:** ^1^Division of Abdominal Cancer, West China Hospital, Sichuan University, Chengdu, Sichuan 610041, China; ^2^School of Clinical Medicine, Chengdu University of Traditional Chinese Medicine, Chengdu, Sichuan 610072, China; ^3^Department of Infectious Diseases, Affiliated Hospital of Chengdu University of Traditional Chinese Medicine, Chengdu, Sichuan 610072, China

## Abstract

This study was designed to investigate the role of a traditional buzui recipe in anti-inebriation treatment. Buzui consists of* Fructus Schisandrae Chinensis*,* Fructus Chebulae*,* Fructus Mume*,* Fructus Crataegi*,* Endothelium Corneum Gigeriae Galli*, and* Excrementum Bombycis*. The buzui mixture was delivered by gavage, and ethanol was delivered subsequent to the final treatment. The effects of buzui on the righting reflex, inebriation rates, and the survival curve are depicted. Blood alcohol concentrations, alanine aminotransferase (ALT) levels, aspartate aminotransferase (AST) levels, and alkaline phosphatase (ALP) levels were recorded. The activities of alcohol dehydrogenase (ADH), aldehyde dehydrogenase (ALDH), and superoxide dismutase (SOD), as well as malonaldehyde (MDA) levels, were also measured. Our results demonstrated that a traditional buzui recipe showed significant effects on promoting wakefulness and the prevention of acute alcohol intoxication, accelerating the metabolism of alcohol in the liver and reducing the oxidative damage caused by acute alcoholism.

## 1. Introduction

Acute alcohol intoxication (AAI) is the result of a single episode of excessive drinking over the oxidative metabolism of the liver. Ethanol accumulates in the body and the brain, causing central nervous system suppression after initial excitement. Clinical manifestations are heterogeneous and involve different organs and apparatuses, with behavioral, cardiac, gastrointestinal, pulmonary, neurological, metabolic, and especially hepatic effects [1]. AAI is a clinically common disease, typically to the detriment of the drinker's health, personal relationships, and social standing [2–4].

The liver is one of the major target organs of ethanol actions [5]. Free radical mechanisms contribute to ethanol-induced liver injury [6]. In general, the production and elimination of oxygen free radicals in the body exist in homeostasis. Hepatocytes have antioxidation enzymes, such as superoxide dismutase (SOD), to mitigate the harm caused by reactive oxygen species and oxidative processes. When this balance is destroyed by alcohol, excessive oxygen free radicals peroxide the biomembrane, causing cell damage and disease [7].

Because of social contact with Western countries and the growing alcohol culture in China, people recognize the dangers of alcohol, but alcoholism remains a persistent social concern. How to prevent and avoid the harm of alcohol when drinking is an outstanding research question. Unfortunately, no effective and safe reagents have yet emerged as AAI preventive drugs. Compared with relatively new synthetic drugs for alcoholic intoxication, traditional Chinese medicine has been used in the prevention and treatment of alcohol-induced liver disease for more than two millennia, and it has significant effects [8]. Moreover, natural medicines present wide therapeutic spectra to various targets and promote the gradual recovery of physical conditions without toxicity. The buzui recipe used in this study derives from the hangover prescription of Professor Xiao-yu Hu and includes* Fructus Schisandrae Chinensis*,* Fructus Chebulae*,* Fructus Mume*,* Fructus Crataegi*,* Endothelium Corneum Gigeriae Galli*, and* Excrementum Bombycis*.

In this study, we investigated the role of this buzui recipe (BZ) in inebriation prevention. RU-21, a classical drug for alcoholic intoxication, was used as the positive control. We present evidence that AAI may cause uncoordinated movements and alterations in hepatocyte structure and functioning and that BZ plays a key role in protecting the liver through antioxidant effects.

## 2. Materials and Methods

### 2.1. Materials and Chemicals

Alcohol metabolism supplement description (RU-21) (lot: 108671), a commonly used hangover drug, was purchased from American Spirit Science Ltd. Detection kits for alanine aminotransferase (ALT) (lot: 120272), aspartate aminotransferase (AST) (lot: 120272), and alkaline phosphatase (ALP) (lot: 120166) were provided by Biosino Biotechnology Co. Ltd. (Beijing, China). Detection kits for superoxide dismutase (SOD) (lot: 20120216), malondialdehyde (MDA) (lot: 20120212), and aldehyde dehydrogenase (ALDH) (lot: 20120214) were obtained from the Nanjing Jiancheng Institute of Biological Engineering (Nanjing, China). The formula for the buzui recipe (BZ, one dose) is presented in Table 1. All ingredients were purchased from the Affiliated Hospital of the Chengdu University of Traditional Chinese Medicine (Chengdu, China) and authenticated by Professor Zhu-yun Yan (Identification Department of the Chengdu University of TCM), according to the* Pharmacopoeia of the People's Republic of China* (2010). Their voucher specimens were deposited at Teaching Hospital of the Chengdu University of Traditional Chinese Medicine (Chengdu, Sichuan, China).

### 2.2. Animals

Two hundred male pathogen-free (SPF) Kunming mice weighing 20 ± 2 g were purchased from the Chengdu Dashuo Experimental Animal Research Center (Chengdu, China). They were housed 3 per cage and maintained at a controlled ambient temperature (23 ± 2°C) under diurnal conditions (light–dark: 07:00–19:00) for one week. All mice were allowed access to laboratory chow and tap water* ad libitum*. The animals were cared for in accordance with* the Guiding Principles for the Care and Use of Animals*.

### 2.3. Dosage of Reagents

After identification by Professor Zhu-yun Yan of the Identification Department of the Chengdu University of TCM of genuine medicinal materials, the herbs were decocted, filtered, and concentrated to 3.5 g·mL^−1^. A gastric lavage dose of 1 kg/mouse = mg/60 kg × 12.33 (*M* refers to the dose of Chinese medicine and 60 kg for adult standard weight) was prepared.

Similarly, an RU-21 adult clinical dose (1.2 g × 6 = 7.2 g/kg/d, equivalent dose mice: 7.2 g/60 kg × 12.33 = 1.48 g/kg/d) was also prepared. Before use, both treatments were ground and then dissolved in NS to make a suspension with a concentration of 106 mg/mL.

### 2.4. Experimental Design

A total of 48 mice were randomly divided into four groups with 12 mice in each group as follows: A, negative control group (Control); B, model group (Model); C, alcohol metabolism supplement description (RU-21) group (RU-21); and D, buzui recipe group (BZ). All mice were fasted on water only for 12 h before the experiment. The RU-21 and BZ groups were dosed with RU-21 1.48 g/kg or BZ 49 g/kg by gavage, respectively. The control and model groups received equal volumes of normal saline instead. Thirty minutes later, apart from the control group given an equal volume of saline, the remainder was dosed with 6.27 g/kg ethanol to establish a mouse model of acute alcoholism.

### 2.5. Estimation of Liver Function

Serum was collected 6 h after modeling. Then, the serum was centrifuged at 3000 r/min for 5 min at 4°C. ALT, AST, and ALP activities in the serum were measured using commercially available detection kits according to the manufacturer's instructions.

### 2.6. Histopathology of the Liver and Pathological Scores

Liver tissues from the right liver were fixed in 10% formalin and embedded with paraffin. Then, 5 *μ*m thick sections were stained with hematoxylin and eosin (H&E). The histopathological changes of the liver were observed with light microscopy.

All slides were read by three investigators who were blinded to the allocation arm of the animal. The investigators were asked to grade the fat variable score and inflammation score in the liver using a semiquantitative scoring system, with zero indicating no discernable injury and 4 indicating the presence of severe injury [9].

### 2.7. Assay of the Metabolism of Ethanol in the Liver

Liver tissue samples from the left liver were prepared by homogenization with ice-cold isotonic saline to yield a 10% (w/v) tissue homogenate. The homogenate was centrifuged at 3000 r/min for 20 min, and the resulting supernatant was stored at −80°C for SOD and MDA estimation. SOD activity was measured by xanthine oxidase, and the MDA content was measured by the presence of thiobarbituric acid reactive substances (TBARS), according to Buege (Buege and Aust 1978 [10]). ADH and ALDH activities in liver were determined by kits according to the manufacturer's instructions.

### 2.8. Blood Ethanol Concentrations

Oxidized forms of nicotinamide-adenine dinucleotide (NAD) were added to the blood standard solution with different concentrations of alcohol. The absorbance of the reaction mixture was recorded at 340 nm, and the regression equation of the ethanol concentration was achieved as* C* = 1237.6*A* − 11.862 (*r* = 0.967) by the standard curve. The ethanol concentration in the blood of mice was calculated by the formula.

### 2.9. Measures of Drunken Behaviors in Mice and Survival Curves

An additional 120 mice were divided into the above four groups with 30 animals per group for observations of drunken behaviors and survival. The treatments were the same as before. The time to loss of righting reflex (alcohol tolerance time) and the times to recovery of the righting reflex (sober-up time) were recorded, and the numbers of deaths over 24 hours were also recorded.

### 2.10. Statistical Analyses

All statistical analyses were performed using SPSS software package version 17.0. Data were expressed as the means ± standard deviation (SD). The significance of the differences between groups was determined using analyses of variance (ANOVA). A value of *P* < 0.05 (2-sided) was considered statistically significant.

## 3. Results

### 3.1. Blood Alcohol Concentrations after Acute Alcoholism Mice

As shown in Table 2, blood alcohol concentrations reached their peak at 2 hours after alcohol intake. Compared with the model group, blood alcohol concentrations were significantly reduced at all times after alcohol administration in both the RU-21 and BZ groups (*P* < 0.01). Furthermore, blood alcohol concentrations were significantly reduced in the animals treated with BZ compared with RU-21 (*P* < 0.01 or *P* < 0.05).

### 3.2. Protective Effects of BZ in Liver Function

**Figure 1:**
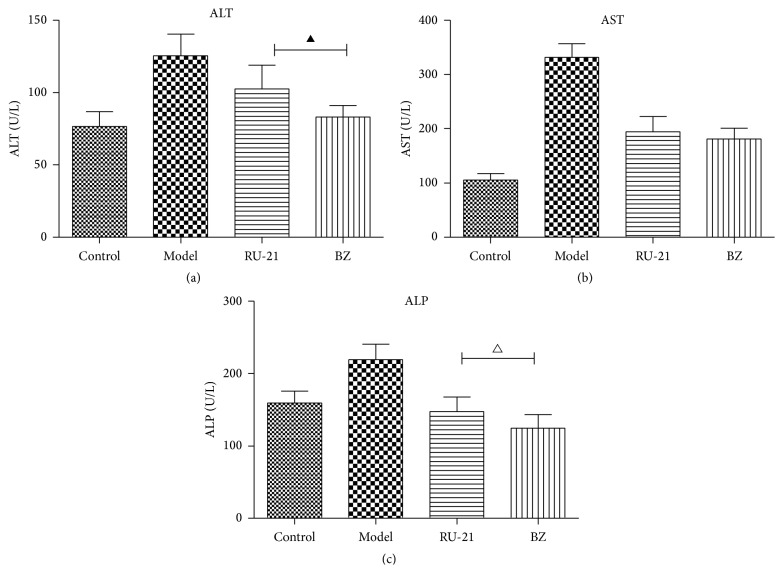
Acute alcohol intoxication in mice liver enzyme changes. (a) ALT; (b) AST; (c) ALP. Control: mice without alcohol or any drugs. Model: the model group treated with alcohol only. RU-21: the positive control group treated with RU-21 before alcohol. BZ: the experiment group treated with buzui recipe before alcohol. ^△^ *P* < 0.05 compared with RU-21 group; ^▲^ *P* < 0.01 compared with RU-21 group.

Serum ALT, AST, and ALP levels are presented in Table 3 and Figure 1. From these results, it was apparent that the ALT, AST, and ALP levels in the RU-21 and BZ groups were decreased compared with those in the model group (*P* < 0.01). Compared with the RU-21 group, the ALT (*P* < 0.01) and ALP levels (*P* < 0.05) of mice in the BZ group were significantly decreased. However, no significant differences were noted between the RU-21 group and the BZ group in AST (*P* > 0.05).

### 3.3. The MDA Content and SOD Activity of Each Group in Liver Tissue

**Figure 2:**
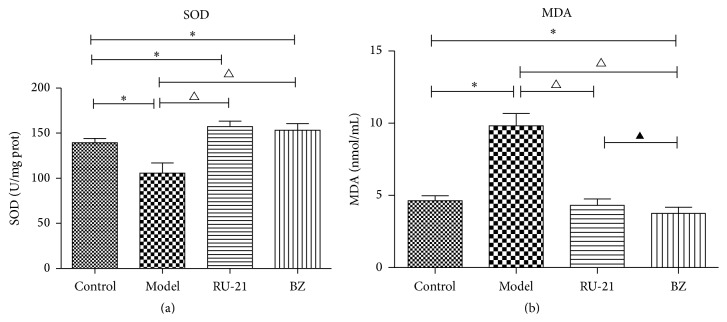
Comparison of acute alcohol intoxication in mice SOD and MDA among groups. (a) SOD; (b) MDA. Control: mice without alcohol or any drugs. Model: the model group treated with alcohol only. RU-21: the positive control group treated with RU-21 before alcohol. BZ: the experiment group treated with buzui recipe before alcohol. For control, model, RU-21, and Buzui groups, the SOD were 139.43 ± 4.85 U/mg prot, 105.67 ± 11.41 U/mg prot, 157.45 ± 5.82 U/mg prot, and 153.26 ± 7.33 U/mg prot, respectively; and the MDA were 4.63 ± 0.35 nmol/mL, 9.82 ± 0.86 nmol/mL, 4.32 ± 0.43 nmol/mL, and 3.75 ± 0.43 nmol/mL, respectively. ^∗^ *P* < 0.01 compared with control group, ^△^ *P* < 0.01 compared with model group, and ^▲^ *P* < 0.05 compared with RU-21 group.

As presented in Table 4 and Figure 2, compared with the control group, SOD activity in the model group was significantly decreased (*P* < 0.01), and MDA levels were significantly elevated (*P* < 0.01). RU-21 and BZ treatment resulted in SOD activity increases and MDA level decreases.

### 3.4. Liver Pathology and Pathological Scores

General observations of samples of the livers in the control group included the presence of a pale red color, smooth surface, no spots or bruising, and softness with no adhesion to the surrounding tissue. The model group was described as dark red, smooth, slightly tough, partial liver swelling, tense and dull liver capsule, and partial surrounding tissue adhesion. The RU-21 and BZ groups were slightly darker than normal mouse livers, exhibiting surfaces without petechiae, with liver capsules that were smooth and shiny, and without adhesion phenomena.

**Figure 3:**
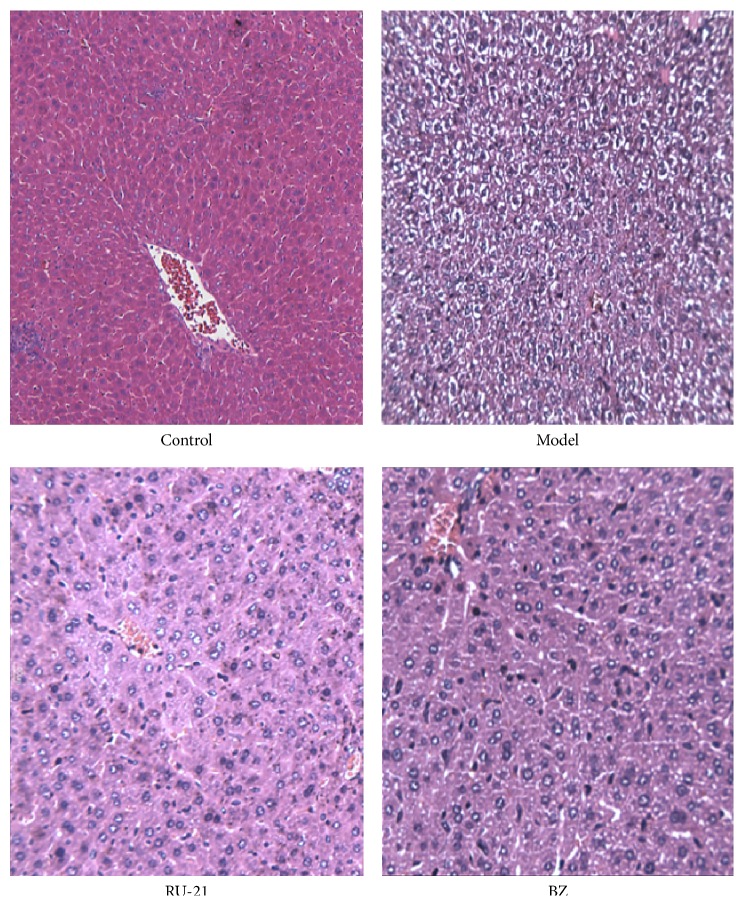
Livers of each group with H&E staining.

In histological examinations using light microscopy, the control group exhibited normal lobular architecture with hepatic cords which are neat and sinusoidal as well as no expansion gap, fatty degeneration, necrosis, or inflammatory cell infiltration. The lobule structures of the livers in the model group were disordered, with swelling obvious around the central vein of the liver cells. The cytoplasm was translucent, exhibiting ballooning degeneration, necrosis, and visible inflammatory cell infiltration. The RU-21 and BZ groups were more comparable to the normal group, with part of the liver cells swollen. In addition, the boundaries between liver cells were clear, and the cytoplasm lightly stained. Individual portal areas for a few inflammatory cells were visible (Figure 3 and Table 5).

### 3.5. ADH and ALDH Levels in Liver Tissue

As shown in Table 6, acute ethanol administered to mice induced incremented ADH activity in the liver, and this elevation in ADH activity in BZ-pretreated animals was considerably increased compared with the model group or the RU-21 group (*P* < 0.01). A similar tendency was also shown for ALDH activity among the model, RU-21, and BZ groups, but the BZ group was lower than the RU-21 group. Pretreatment with BZ further enhanced ADH and ALDH activities in the liver after alcohol administration.

### 3.6. Effects on the Behavior of Mice

**Figure 4:**
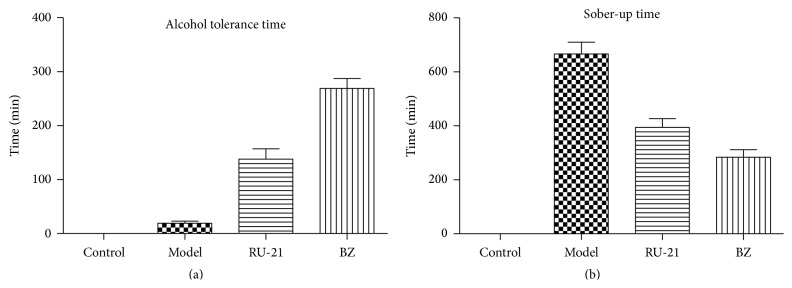
Comparison of alcohol tolerance time and sober-up time among groups. (a) Alcohol tolerance time; (b) sober-up time.

Compared with the model group, the alcohol tolerance times of mice in the RU-21 and BZ groups were significantly extended. Similarly, the sober-up time was shortened. Significant differences were noted between the RU-21 and BZ groups, indicating that BZ had stronger effects (Figure 4, Table 7). We also observed changes in mouse appetite, behavior, and various symptoms, such as urinary incontinence, lethargy, irritability, and convulsion. These behaviors were common in the model group but rare in the BZ group.

### 3.7. Survival Curves

**Figure 5:**
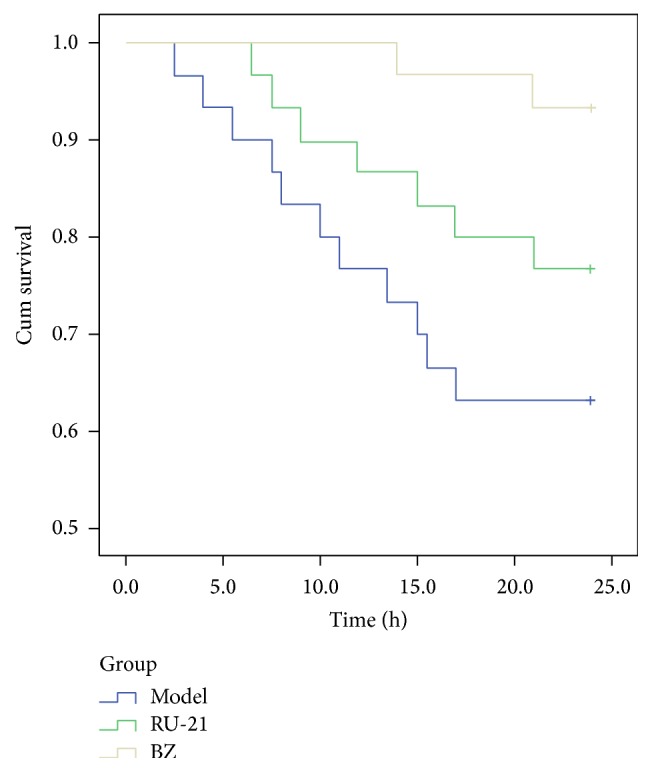
The survival curve. Model: the model group treated with alcohol only. RU-21: the positive control group treated with RU-21 before alcohol. BZ: the experiment group treated with buzui recipe before alcohol.

We excluded the control group from our survival curves because there were no deaths at the endpoint. BZ significantly improved survival rates after acute alcohol intoxication (Figure 5).

## 4. Discussion

In this study, we observed increased serum ALT, AST, and ALP levels and reductions in the uncoordinated movements of mice and damage to their livers after acute alcohol intoxication models. RU-21 and BZ both ameliorated liver function and prevented AAI; however, the effects of BZ were stronger.

Currently, oral gavage and intraperitoneal injection are the main two methods to dose ethanol for acute alcohol intoxication in mice. We choose gavage because it simulates normal human drinking, and the alcohol is absorbed through the digestive tracts of the mice. Furthermore, alcohol administered intragastrically results in obvious abnormal liver functioning even compared with similar pathologic changes in the human liver following acute intoxication [11].

We performed prior experiments (data not shown) in accordance with the related literature using ethanol at doses of 4.48 g/Kg, 5.38/Kg, 6.27 g/Kg, 7.17 g/Kg, and 8.06 g/Kg. We observed drunken mice and computed intoxication rates and mortality at each of these doses. After repeated tests, we found that administration in the amount of 6.27 g/kg results in inebriation rates ≥90% and mortality rates ≤40%, reflecting modeling success. We determined that the mouse became inebriated when the righting reflex disappeared.

The efficiency of traditional Chinese medicine (TCM) is based on reinforcing an organism's natural healing power and ability to restore energy homoeostasis. Previous studies have demonstrated that a likely mechanism for at least some of these activities is interaction with redox balance and the prevention of oxidative stress [12]. Some traditional Chinese medicinal seeds and fruits are well known for their antioxidant properties. Regarding the Chinese medicine included in this recipe,* Fructus Chebulae* might be the mechanism for the inhibition of oxidative damage [13].* Fructus Schisandrae Chinensis* exhibits pharmacological effects, including liver protection, antihypoxia, antifatigue, immunity enhancement, antioxidation, and antitumor [14, 15].* Fructus Mume* enhances intestinal propulsive motion and motility [16], and it might play a therapeutic role by enhancing SOD activity and lowering MDA content [17].* Fructus Crataegi* might regulate lipid metabolism, enhancing antioxidant properties and reducing the release of inflammatory factors [18].* Endothelium Corneum Gigeriae Galli* possesses strong hydroxyl radical scavenging properties as well as Fe^2+^ chelating and lipid peroxidation inhibitory and antioxidant activities* in vitro* [19]. Extracts from* Excrementum Bombycis* (BE), which consists of various bioactive constituents and mulberry leaves (the preferred food of silkworms), possess anti-inflammatory, antidiabetic, and antioxidative effects [20]. BZ combines these Chinese herb medicines following the principle of clearing heat and resolving stasis. BZ exhibited a positive effect in this study.

Over recent years, studies have extensively searched for effective herbs for the treatment of alcohol-related diseases. BZ is derived from some uncommon herbs that exhibit anti-inebriation effects, including* Flos Puerariae* and* Radix Puerariae*. Its formulation is based on clinically reliable alcohol metabolism pathways, and the recipe refers to TCM theory, matching herbs to improve their natural hepatoprotective effects. BZ is innovative and, more importantly, no treatment-related side effects were discovered.

In the present study, we found that buzui has positive effects, and we believe it may even have more comprehensive effects in human beings, including postponing the inebriation time, reducing sleep duration, reducing the degree of inebriation, and exhibiting prominent effects against liver oxidative damage.

## 5. Conclusion

Our buzui recipe demonstrated significant effects on promoting wakefulness and preventing acute alcohol intoxication. This mixture accelerated the metabolism of alcohol in the liver and reduced the oxidative damage caused by acute alcoholism. These effects are thought to be mediated by interactions with redox balance and the prevention of oxidative stress.

## Figures and Tables

**Table 1:** The formula for the buzui recipe (BZ, one dose).

Chinese medicine name	Medicinal parts	Origin	Amount in preparation (g)
*Schisandra chinensis *(Turcz.)Baill. (Beiwuweizi)	Fruit	Heilongjiang province	40

*Terminalia chebula *Retz. (Hezi)	Fruit	Yunnan province	40

*Dark plum fruit* (Wumei)	Fruit	Sichuan province	50

*Crataegus pinnatifida *Bunge (shanzha)	Fruit	Shandong province	40

Chicken's gizzard membrane (Jineijin)	Gizzard	Sichuan province	60

Silkworm excrement (cansha)	Excrement	Sichuan province	30

**Table 2:** Blood alcohol concentrations following acute alcoholism mice (unit: mg·mL^−1^).

Hour	Groups			
	Control	Model	RU-21	BZ
0 h	0	0	0	0
0.5 h	0	36.03 ± 0.79	30.33 ± 0.67^*∗*^	28.16 ± 1.09^**∗**▲^
1 h	0	37.22 ± 0.27	32.28 ± 0.7^*∗*^	31.12 ± 0.95^**∗**▲^
1.5 h	0	39.82 ± 0.69	34.34 ± 0.83^*∗*^	32.74 ± 0.97^**∗**▲^
2 h	0	45.03 ± 0.73	39.6 ± 0.52^*∗*^	37.47 ± 1.04^**∗**▲^
3 h	0	38.7 ± 0.55	30.96 ± 0.67^*∗*^	30.02 ± 0.86^**∗**▲^
6 h	0	33.13 ± 0.46	28.36 ± 1.36^*∗*^	26.98 ± 1.17^**∗**△^

^*∗*^ *P* < 0.01 compared with model group, ^△^ *P* < 0.05 compared with RU-21 group, and ^▲^ *P* < 0.01 compared with RU-21 group.

**Table 3:** Comparisons of acute alcohol intoxication in mice: ALT, AST, and ALP levels in the groups ($\bar{x}\pm S$, *n* = 12).

Groups	ALT (U/L)	AST (U/L)	ALP (U/L)
Control	76.58 ± 10.34	105.17 ± 12.34	159.58 ± 16.43
Model	125.56 ± 14.89^*∗*^	331.11 ± 25.43^*∗*^	219.44 ± 21.38^*∗*^
RU-21	102.40 ± 16.50^*∗*^	194.50 ± 28.15^*∗*^	147.30 ± 20.20^*∗*^
BZ	83.09 ± 8.12^*∗*▲^	180.73 ± 20.35^*∗*^	124.91 ± 18.78^*∗*△^

^*∗*^ *P* < 0.01 compared with control group, ^△^ *P* < 0.05 compared with RU-21 group, and ^▲^ *P* < 0.01 compared with RU-21 group.

**Table 4:** Comparisons of Acute alcohol intoxication in mice: SOD and MDA levels in the groups ($\bar{x}\pm S$, *n* = 12).

Groups	SOD (U/mg prot)	MDA (nmol/mL)
Control	139.43 ± 4.85	4.63 ± 0.35
Model	105.67 ± 11.41^*∗*^	9.82 ± 0.86^*∗*^
RU-21	157.45 ± 5.82^*∗*△^	4.32 ± 0.43^△^
BZ	153.26 ± 7.33^*∗*△^	3.75 ± 0.43^*∗*▲△^

^*∗*^ *P* < 0.01 compared with Control group, ^△^ *P* < 0.01 compared with Model group, ^▲^ *P* < 0.05 compared with RU-21 group.

**Table 5:** Pathological scores ($\bar{x}\pm S$, *n* = 12).

Groups	Fat variable score	Inflammation score
Control	0	0
Model	3.17 ± 1.03^*∗∗*^	2.67 ± 0.89^*∗∗*^
RU-21	2.17 ± 1.27^#^	2.33 ± 0.89
BZ	1.33 ± 1.51^##△^	1.58 ± 0.79^##▲^

^*∗∗*^ *P* < 0.01 compared with control group, ^#^ *P* < 0.05 compared with model group, ^##^ *P* < 0.01 compared with model group, ^△^ *P* < 0.05 compared with RU-21 group, and ^▲^ *P* < 0.05 compared with RU-21 group.

**Table 6:** Comparisons of acute alcohol intoxication in mice: ADH and ALDH levels in the groups ($\bar{x}\pm S$, *n* = 12).

Groups	ADH (nmol/(g·min))	ALDH (U/L)
Normal	0.175 ± 0.016	21.27 ± 3.71
Model	0.191 ± 0.016	46.04 ± 8.03^*∗*^
RU-21	0.213 ± 0.015^*∗*△^	124.46 ± 27.68^*∗*△△^
BZ	0.295 ± 0.037^*∗*△△▲▲^	93.73 ± 15.37^*∗*△△▲^

^*∗*^ *P* < 0.05 compared with control group, ^△^ *P* < 0.05, ^△△^ *P* < 0.01 compared with model group, and ^▲^ *P* < 0.05, ^▲▲^ *P* < 0.01 compared with RU-21 group.

**Table 7:** Comparisons of the sober-up times and death rates among groups ($\bar{x}\pm S$, *n* = 30).

Groups	*N*	Sober-up time ($\bar{x}\pm S$, min)	Death numbers or rate (*n*, %)
Normal	30	—	—
Model	19	666.2 ± 44.31	11 (37)
RU-21	23	395.2 ± 31.95^*∗*^	7 (23)
BZ	28	284.40 ± 27.0^*∗*△^	2 (6)

^*∗*^ *P* < 0.01 compared with model group and ^△^ *P* < 0.01 compared with RU-21 group.

## References

[1] Vonghia L., Leggio L., Ferrulli A., Bertini M., Gasbarrini G., Addolorato G. (2008). Acute alcohol intoxication. *European Journal of Internal Medicine*.

[2] Brewer R. D., Swahn M. H. (2005). Binge drinking and violence. *The Journal of the American Medical Association*.

[3] American Psychiatric Association (2000). *Diagnostic and Statistical Manual of Mental Disorders*.

[4] Sindelar H. A., Barnett N. P., Spirito A. (2004). Adolescent alcohol use and injury. *Minerva Pediatrica*.

[5] Lieber C. S. (1991). Hepatic, metabolic and toxic effects of ethanol: 1991 update. *Alcoholism: Clinical and Experimental Research*.

[6] Lieber C. S. (2001). Alcoholic liver injury: pathogenesis and therapy in 2001. *Pathologie Biologie*.

[7] Beier J. I., McClain C. J. (2010). Mechanisms and cell signaling in alcoholic liver disease. *Biological Chemistry*.

[8] Xu B. J., Zheng Y. N., Sung C. K. (2005). Natural medicines for alcoholism treatment: a review. *Drug and Alcohol Review*.

[9] Fatty Liver and Alcoholic Liver Disease Group of the Chinese Medical Association (2010). The diagnosis and treatment of alcoholic liver disease. *Chinese Journal of Liver Diseases*.

[10] Buege J. A., Aust S. D. (1978). Microsomal lipid peroxidation. *Methods in Enzymology*.

[11] Jinbo Z., Tailing W., Jing Z. (1996). Rat model of acute alcoholic liver injury. *Journal of China-Japan Friendship Hospital*.

[12] Matkowski A., Jamiołkowska-Kozlowska W., Nawrot I. (2013). Chinese medicinal herbs as source of antioxidant compounds—where tradition meets the future. *Current Medicinal Chemistry*.

[13] Gaire B. P., Kim H. (2014). Neuroprotective effects of Fructus Chebulae extracts on experimental models of cerebral ischemia. *Journal of Traditional Chinese Medicine*.

[14] Chen W., Ji Y. (2007). Progress on pharmacological action of fructus schisandrae polysaccharide. *Food and Drug*.

[15] Wagner H., Bauer R., Melchart D., Xiao P.-G., Staudinger A. (2011). Fructus schisandrae—*Wuweizi*. *Chromatographic Fingerprint Analysis of Herbal Medicines*.

[16] Wang L., Zhang H. Y. (2010). Comparison of pharmacological effects of Fructus Mume and its processed products. *Zhong Yao Cai*.

[17] He A., Wang Y., Lin S. (2012). The effect of Fructus Mume water extract on experimental ulcerative colitis in mice. *Journal of Pharmaceutical Practice*.

[18] Wang W., Yang B., Wang L. (2011). Antiatherogenic effect of radix *Salvia miltiorrhiza* and Fructus Crataegi on experimental atherosclerosis in rats. *China Journal of Chinese Materia Medica*.

[19] Xiong Q., Li X., Zhou R. (2014). Extraction, characterization and antioxidant activities of polysaccharides from *E. corneum gigeriae galli*. *Carbohydrate Polymers*.

[20] Moon M., Choi J. G., Kim S. Y. (2014). Bombycis excrementum reduces amyloid-*β* oligomer-induced memory impairments, neurodegeneration, and neuroinflammation in mice. *Journal of Alzheimer's Disease*.

